# Enteral administration of alectinib for ALK-positive non-small cell lung cancer in an elderly patient: A case report

**DOI:** 10.1097/MD.0000000000027611

**Published:** 2021-10-29

**Authors:** Quentin Dominique Thomas, Marie Pautas, Marie-Noëlle Guilhaume, Fréderic Fiteni, Tony Ge, Nicolas Girard

**Affiliations:** a Medical Oncology Department, Institut du Cancer de Montpellier (ICM), Montpellier University, Montpellier, France; b Medical Oncology Department, Institut Curie, Paris, France; c Paris Saclay University, Saint Aubin, France; d Medical Oncology Department, Centre Hospitalier Universitaire de Nîmes, Nîmes, France.

**Keywords:** alectinib, case report, elderly patient, enteral administration, non-small cell lung cancer

## Abstract

**Rationale:**

Alectinib is a tyrosine kinase inhibitor (TKI) approved for use as first-line metastatic therapy for patients with anaplastic lymphoma kinase-rearranged non-small cell lung cancer. Certain medical conditions related to the tumor lesions may not allow oral administration of TKIs.

**Patient concerns:**

We hereby report the case of a 90-year-old patient with anaplasic lymphoma kinase-rearranged lung cancer with severely impaired general condition and swallowing disorders.

**Diagnosis:**

A thoracic computerized tomography (CT)-scan confirmed the presence of a mediastinal tumor lesion explaining the swallowing disorders secondary to recurrent paralysis.

**Interventions:**

As no oral administration was feasible, alectinib was administered by percutaneous gastrostomy.

**Outcomes:**

The patient had few side-effects. He presented a major clinical and radiological response. After 2 months of treatment with alectinib, his mini-mental state examination had increased from 8/30 to 23/30. He had a 60% reduction in targeted pulmonary, bone and node lesions according to the Response Evaluation Criteria in Solid Tumors version 1.1 (RECIST 1.1). After 6 months of treatment, the patient's performance status had evolved from 3 to 1. This improvement in general condition made it possible to remove the feeding tube.

**Lessons:**

In cases of lung cancer with oncogenic addiction, enteral administration of TKIs should be considered for elderly patients with an impaired general condition.

## 1 Introduction

Historically, the treatment of metastatic non-small cell lung cancer (NSCLC) has been based on cytotoxic chemotherapy. Immunotherapy and tyrosine kinase inhibitors (TKIs) have increased the therapeutic opportunities.^[1]^ Anaplastic lymphoma kinase (ALK) rearrangement is present in approximately 5% of metastatic NSCLC.^[2]^ ALK positive patients benefit from TKIs, such as alectinib, a specific second-generation ALK TKI. Alectinib has shown a progression-free survival of 34.8 months compared with 10.9 months with crizotinib, the historical first-line treatment for ALK positive patients (stratified hazard ratio = 0.43; 95% confidence interval: 0.32–0.58).^[3]^ Oral administration of TKIs may not be feasible in certain medical situations such as respiratory failure or swallowing difficulties due to carcinomatous meningitis or mediastinal lymph node involvement. Enteral administration of TKIs may thus be the only available resource.

Here we present the case of a 90-year-old patient with swallowing disorders, for whom alectinib was administered by percutaneous gastrostomy with a major therapeutic response, and improved quality of life.

## 2 Case presentation

This case report concerns a 90-year-old male patient who had never smoked, with a medical history of hypertension, atrial fibrillation treated by oral anticoagulants, hypothyroidism induced by the use of amiodarone, and a localized prostate cancer treated by surgery 20 years ago.

Before the onset of symptoms, the patient had complete autonomy (6/6) on the activities of daily living (ADL) scale, and 8/8 on the instrumental ADL scale. He used to walk every day and, as a retired professor of biology, even worked from time to time.

His main symptoms, which had appeared in less than a month, were a serious deterioration in general condition including anorexia, weight loss, asthenia, and psychomotor slowing. This had resulted in a serious loss of autonomy with a performance status (Eastern Cooperative Oncology Group Performance Status [ECOG PS]) of 3, instrumental ADL of 0/6, an ADL of 2/6, and a mini-mental state examination of 8/30. He had also been suffering from a cough, dyspnea requiring oxygen therapy, and several episodes of hemoptysis for 2 weeks before diagnosis. The latest symptoms were major swallowing disorders and dysphonia in the last week before diagnosis.

A computerized tomography (CT) scan showed a mediastino-hilar mass in the left lung associated with unilateral pleural effusion. A positron emission tomography scan showed hypermetabolism of the left pulmonary mass and pleural effusion, contralateral carcinomatous lymphangitis, multiple hypermetabolic sus-diaphragmatic lymphadenopathies, bilateral adrenal metastasis and, finally, multiple bone lesions in the axial skeleton. Magnetic resonance imaging of the brain excluded brain metastasis. Swallowing disorders were certainly related to compression of the left recurrent nerve by a lymphadenopathy recorded on the CT-scan. Biopsies made during a bronchoscopy led to the diagnosis of an ALK-positive pulmonary adenocarcinoma (ALK rearrangement detected by immunohistochemistry). The final staging was cT4N3M1c (stage IVb) according to the 8th version of the TNM classification.

The patient was treated with alectinib administrated twice daily (600 mg × 2, i.e., 4 150 mg capsules morning and evening). Because of the patient's swallowing disorders, oral administration of treatments and feeding were impossible. It was thus decided to administer alectinib via a nasogastric feeding tube by opening the capsules and dissolving the drug in water, despite the fact that there were no manufacturer's instructions for enteral use of this drug. Due to episodes of confusion, the patient removed the feeding tube several times. A collegial decision was made to perform percutaneous gastrostomy despite the patient's advanced age, medical history, and the presence of cognitive disorders. Percutaneous gastrostomy was thus used to administer alectinib and other drugs (apart from anticoagulants) and also for enteral nutrition and hydration.

**Figure 1:**
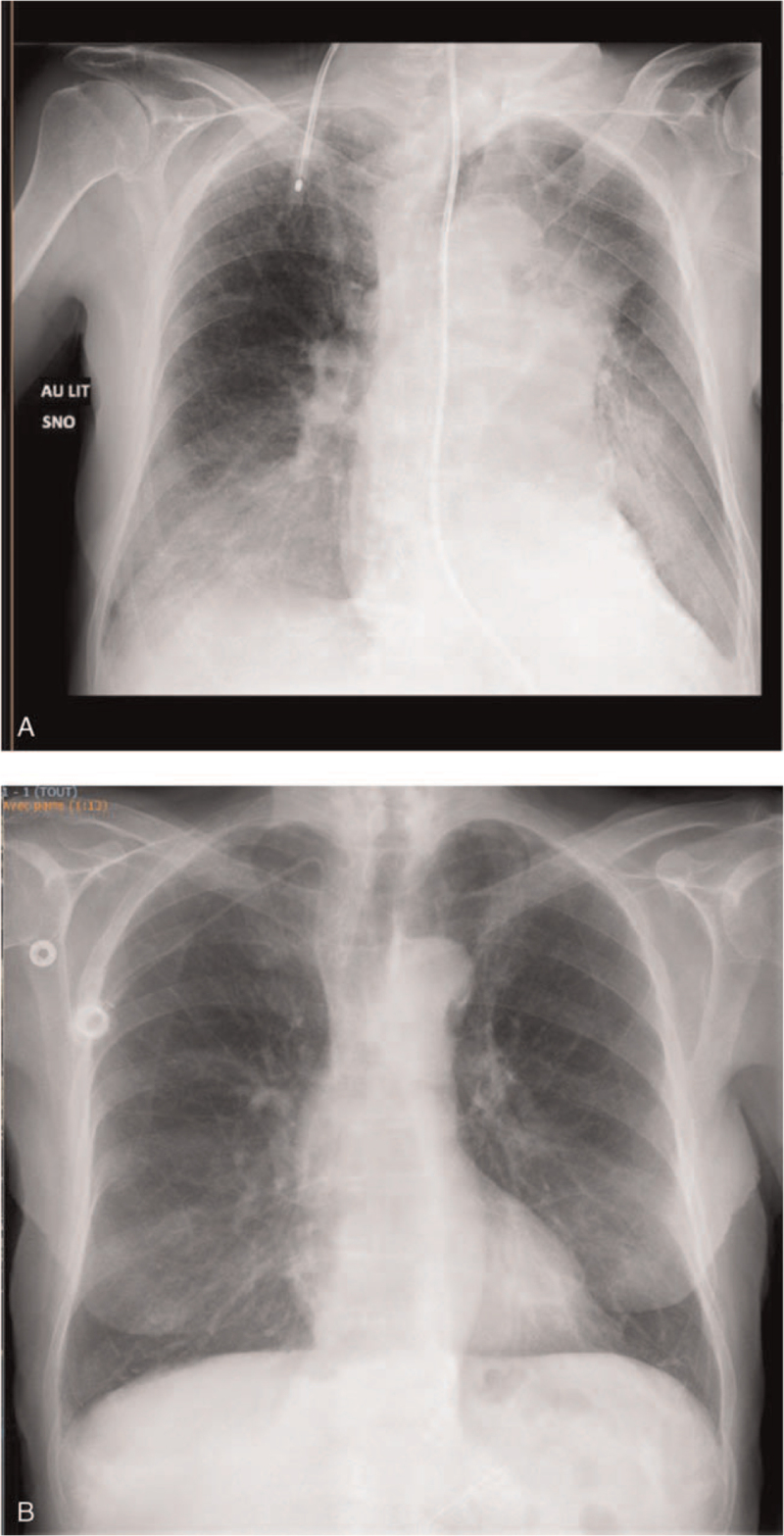
Tumor assessment by chest X-ray at baseline (A) and after 3 months of treatment with alectinib (B).

**Figure 2:**
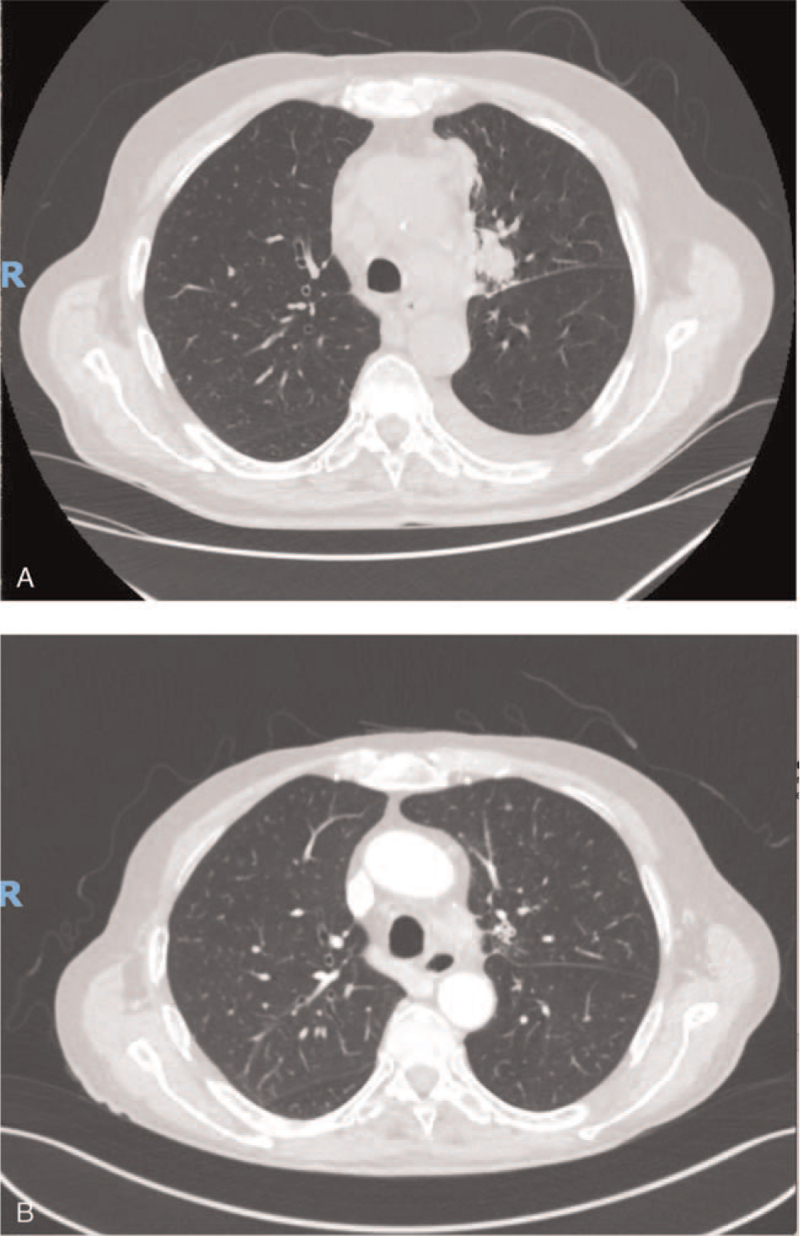
Tumor assessment by CT-scan at baseline (A) and after 6 months of treatment with alectinib (B). CT = computerized tomography.

Enteral nutrition and systemic treatment by alectinib were well tolerated except for grade 2 hepatic cytolysis (according to common terminology criteria for adverse events [CTCAE] v5.0) which appeared after 10 days of treatment with a spontaneous decrease after 2 weeks. The treatment had important clinical benefits for the patient such has oxygen weaning within the first 2 weeks of treatment, an improvement in cognitive disorders (mini-mental state examination 23/30 after 2 months of treatment), improvements in the patient's general condition, and recovery of his autonomy (ECOG PS 1, ADL 4,5/6, Instrumental ADL 2/6 after 4 months of treatment). Imaging showed a partial response on pulmonary, bone and node lesions, according to version 1.1 of the Response Evaluation Criteria in Solid Tumors (RECIST 1.1). Total tumor regression was 28%, 37%, and 60% compared with the first baseline CT-scan after 1, 3, and 6 months of treatment respectively (Figs. 1 and 2). The improvement in the patient's general condition and disappearance of swallowing disorders, confirmed by a swallowing test given by a physiotherapist, allowed us to remove the percutaneous gastrostomy after 6 months of alectinib. After 10 months’ follow-up, the patient is now in excellent physical condition and continues to take alectinib orally with no recurrence of the swallowing disorders.

## 3 Discussion

We hereby report the case of a patient with ALK-rearranged metastatic lung adenocarcinoma who presented with partial response to enteral administration of alectinib. There are numerous case reports arguing for the efficacy of enteral administration of TKIs in oncogene-addicted lung cancer. One review of the literature published in 2018 identified 14 cases of patients treated with TKIs through nasogastric tube or percutaneous gastrostomy mainly due to respiratory failure or swallowing disorders.^[4]^ The median age in that population was 59.5 years (range 36–81 years). Interestingly, all 14 patients had a positive clinical response and almost all responded within 1 to 2 weeks after starting treatment. In addition, 4 patients were able to shift over to oral TKI intake after a few weeks of treatment.

As mentioned above, our patient was diagnosed with an altered general condition, ECOG PS 3. In the ALEX trial, the study population comprised 93% ECOG PS 0–1 patients, 7% ECOG PS 2 patients, and no ECOG PS 3–4 patients. The median age of patients treated with alectinib was 58 years (25–88) with limited information dedicated to elderly patients.^[5]^ A real-world surveillance study conducted in Japan reported data from 1251 patients treated with alectinib. ECOG PS 3 patients had a poorer overall survival rate at 18 months than patients with an intermediate (ECOG PS 2) or good (ECOG PS 0–1) general condition with survival rates of 27.2%, 44.5%, and 83.7% respectively. However, there was no report of increased toxicity for ECOG PS ≥2 versus ECOG PS ≤1 patients. Furthermore, patients over 75 years of age (12.9%) had similar survival curves to younger populations, suggesting that alectinib may be proposed to patients regardless of age.^[6]^

Like other TKIs, alectinib is administered at a fixed dose. One limitation of our case report is that we did not perform a pharmacokinetic blood test to assess whether the patient was within the therapeutic window. Pharmacokinetic data suggest that alectinib has high interindividual variability of 40% to 45% in pharmacokinetic exposure.^[7]^ Recent data have shown that, in patients receiving alectinib, the median progression- free survival is significantly shorter in patients with plasma levels below the minimum plasma concentration (Cmin = 435 ng/mL). Therefore, therapeutic drug monitoring should be part of routine clinical management for these agents.^[8]^ However, there are indirect arguments against underdosing in the present case. Indeed, our patient presented side effects (hepatic cytolysis) as well as the rapid therapeutic response objectified on the chest X-ray.

Certain studies on pharmacokinetic differences in the use of oral and direct enteral administration of drugs (nasogastric tube or gastrostomy),^[9,10]^ show that there are no significant difference between these 2 routes, either in terms of efficacy, plasma concentration, or adverse effects. Stability in water and acidic pH are key factors in such a setting. It has been suggested that alectinib is stable in iced water, water at room temperature, and water at 37 °C for 24 hours.^[11]^ As the capsule is not gastro-resistant, there should be no differences in the impact of gastric acidity between the 2 administration routes. Indeed, gastric acidity actually increases the solubility of alectinib which enhances its bioavailability.^[12]^

Our case report is fairly unique in that our patient was elderly and had an altered ECOG PS which did not plead for the administration of systemic treatment. It also reinforces the necessity for systematic screening of metastatic patients with NSCLC for targetable molecular alterations.^[13]^ We believe that invasive management allowing the enteral administration of TKI (nasogastric tube or percutaneous gastrostomy) should be considered, whatever the general condition or age of patients with oncogenic addiction for NSCLC.

## 4 Patient consent statement

The patient has provided informed consent for the publication of this case.

## Acknowledgments

The authors wish to thank the patient and his family for allowing them to present this case report.

They also wish to thank Teresa Sawyers, Medical Writer at the BESPIM, Nîmes University Hospital, for revising this manuscript.

## Author contributions

**Conceptualization:** Quentin Dominique Thomas, Marie Pautas, Nicolas Girard.

**Investigation:** Tony Ge.

**Resources:** Marie Pautas.

**Supervision:** Marie-Noëlle Guilhaume, Frederic Fiteni, Nicolas Girard.

**Writing – original draft:** Quentin Dominique Thomas, Marie Pautas, Tony Ge.

**Writing – review & editing:** Quentin Dominique Thomas, Marie-Noëlle Guilhaume, Frederic Fiteni, Nicolas Girard.
